# Abnormal connectivity between the default mode and the visual system underlies the manifestation of visual hallucinations in Parkinson’s disease: a task-based fMRI study

**DOI:** 10.1038/npjparkd.2015.3

**Published:** 2015-04-22

**Authors:** James M Shine, Alana J Muller, Claire O’Callaghan, Michael Hornberger, Glenda M Halliday, Simon JG Lewis

**Affiliations:** 1 Brain and Mind Research Institute, The University of Sydney, Sydney, NSW, Australia; 2 School of Psychology, Stanford University, Palo Alto, CA, USA; 3 Neuroscience Research Australia and The University of New South Wales, Randwick, NSW, Australia; 4 Department of Clinical Neurosciences, University of Cambridge, Cambridge, UK

## Abstract

**Background:**

The neural substrates of visual hallucinations remain an enigma, due primarily to the difficulties associated with directly interrogating the brain during hallucinatory episodes.

**Aims:**

To delineate the functional patterns of brain network activity and connectivity underlying visual hallucinations in Parkinson’s disease.

**Methods:**

In this study, we combined functional magnetic resonance imaging (MRI) with a behavioral task capable of eliciting visual misperceptions, a confirmed surrogate for visual hallucinations, in 35 patients with idiopathic Parkinson’s disease. We then applied an independent component analysis to extract time series information for large-scale neuronal networks that have been previously implicated in the pathophysiology of visual hallucinations. These data were subjected to a task-based functional connectivity analysis, thus providing the first objective description of the neural activity and connectivity during visual hallucinations in patients with Parkinson’s disease.

**Results:**

Correct performance of the task was associated with increased activity in primary visual regions; however, during visual misperceptions, this same visual network became actively coupled with the default mode network (DMN). Further, the frequency of misperception errors on the task was positively correlated with the strength of connectivity between these two systems, as well as with decreased activity in the dorsal attention network (DAN), and with impaired connectivity between the DAN and the DMNs, and ventral attention networks. Finally, each of the network abnormalities identified in our analysis were significantly correlated with two independent clinical measures of hallucination severity.

**Conclusions:**

Together, these results provide evidence that visual hallucinations are due to increased engagement of the DMN with the primary visual system, and emphasize the role of dysfunctional engagement of attentional networks in the pathophysiology of hallucinations.

## Introduction

Theoretical models have implicated sensory, attentional, and cognitive deficits in the development of visual hallucinations;^1–7^ however, empirical evidence remains elusive, owing mainly to the obstacles inherent in eliciting hallucinatory phenomena experimentally. As such, the neural mechanisms underpinning hallucinations remain poorly defined, particularly in neurodegenerative diseases such as Parkinson’s disease. Although most studies investigating hallucinations have been undertaken in psychiatric populations, e.g., in schizophrenia,^2^ abnormal perceptual experiences are remarkably common in Parkinson’s disease, suggesting that Parkinson’s disease may represent an important model for probing visual hallucinations. Despite this potential utility, initial strategies to investigate hallucinations in Parkinson’s disease have been reliant on either correlating brain activity with self-reported hallucinations^8–11^ or interrogating impaired performance on basic visuoperceptual tasks.^12,13^ Although such measures have provided insights into the pathophysiology of hallucinations, the utility of these approaches is limited by a lack of objective and concurrent assessment of the hallucinating brain.

The development of the Bistable Percept Paradigm (BPP) task^1,14^ (Table 1) has circumvented many of these assessment issues, as the task is capable of reproducibly eliciting visual hallucinatory phenomena (“misperceptions”) in susceptible individuals.^1^ The BPP requires participants to view a series of monochromatic images that contain either a “stable” or “bistable” image, the latter associated with multiple perceptual interpretations (e.g., Table 1).^1,14^ Patients with visual hallucinations regularly misperceive additional features within “stable” stimuli that contain only a single image.^1,14^ That is, they see something hidden in an image that is not there—the very definition of a hallucination. Importantly, the misperceptions elicited by the BPP only rarely occur in individuals without clinically evident hallucinations,^1^ highlighting the utility of the paradigm as a highly specific, objective marker of visual hallucinations. Previously, we have hypothesized that, in the presence of impaired exogenous attentional network function, increased activity within endogenous attentional networks could potentially manifest as aberrant visual perceptual experiences in Parkinson’s disease patients with hallucinations.^4,15^ However, no study to date has utilized the BPP, or any other objective assessment task, to determine the functional correlates of such visual misperceptions in a susceptible clinical population.

In this study, we exploited the utility of the BPP task to elicit visual misperceptions in 35 individuals with idiopathic Parkinson’s disease and examined the neural correlates of these episodes using functional magnetic resonance imaging (fMRI). We first compared the neural activation during the normal visual perception of monochromatic images in two well-matched groups of Parkinson’s disease patients, who differed only in their experience of self-reported hallucinatory symptoms. Then, by utilizing a novel method for the estimation of task-based functional connectivity, we explored the patterns of neural network activity and connectivity during overt visual misperceptions. We hypothesized that the “misperception” of images on the BPP would be reflected by impairments in networks responsible for exogenous attention, leading to an over-reliance on endogenous attentional networks for perception. Further, we also hypothesized that any network abnormalities detected during misperceptions would be reflected across a clinical spectrum, relating not only to both abnormal performance on the BPP but also to independent clinical markers of visual hallucinations.

## Materials and methods

### Participants

Thirty-five patients with idiopathic Parkinson’s disease were recruited from the Parkinson’s Disease Research Clinic at the Brain and Mind Research Institute. All patients satisfied the UKPDS Brain Bank criteria and displayed no overt signs of dementia.^16^ Permission for the study was obtained from the local research ethics committee and all patients gave written informed consent.

### Neurological and neuropsychological assessments

All patients underwent assessment in their “on” state, were rated as between Hoehn and Yahr stages I–III, and were assessed on the Unified Parkinson’s Disease Rating Scale (UPDRS; Table 2).^17^ Neither group displayed visual deficits as measured by the Pelli–Robson contrast sensitivity test,^18^ and subjects were allowed to wear corrective lenses during the experiment. The Montreal Cognitive Assessment was used as a general measure of cognition,^8^ and the Beck Depression Inventory-II determined the presence of affective disturbance.^19^ Dopaminergic dose equivalence scores were also calculated for each patient.^20^ To assess for the presence of clinically identified hallucinations, all participants were assessed according to the second question of the Movement Disorders Society-UPDRS (“Over the past week have you seen things that were not really there?”), as well as on the Scales for Outcome in Parkinson’s disease–Psychiatric Complications (SCOPA–PC_1–4_).^21^

### Bistable percept paradigm

Each patient performed the BPP^1,14,15^ while lying recumbent in a 3-T MRI scanner (General Electric, General Electric, NSW, Australia). The BPP is a computer-based task that requires participants to evaluate a randomized battery of 40 monochromatic “monostable” and 40 “bistable” images (Table 1). Participants were required to classify a series of images as either “single” (capable of only one perceptual interpretation) or “hidden” (a bistable percept, capable of more than one perceptual interpretation). Participants had up to 10 s to evaluate each image; however, they could respond before the time limit if they were confident. A practice session using unique images was administered containing examples of 10 stable and bistable images.

In the scanner, each experimental trial consisted of the presentation of a crosshair (variable duration: 0.2–1.0 s) after which an image was randomly presented. If the patient responded within 10 s, then the next trial would begin. If no response was made within the 10-s window, then a screen would appear to prompt a decision. Each response was recorded during the scanning session by using a two-button “response” box (left: “stable”; right: “bistable”). Immediately following the scanning session, a manipulation check was performed and only those images with consistent answers were included in the final analysis.

For each experimental trial, the responses of participants were scored as either (i) a correct image—in which a participant correctly identified an image; (ii) a missed image—in which a participant misclassified a bistable image as single; or (iii) a misperception—whereby a participant incorrectly classified a stable image as bistable (Table 1). In the first experiment, we compared neural activity between hallucinators and non-hallucinators on correct stable items. In the second experiment, we separately assessed the 21 hallucinators (those individuals with misperception rates greater than a previously defined cut score of 11%; Shine *et al.*,^1^) to directly compare BOLD signal patterns between misperceptions (stable image identified as bistable) with correctly interpreted single images, allowing an estimate of the neuronal correlates implicated in the evolution of the hallucinatory state. In keeping with previous studies,^1–7,14,22^ we also used each patient’s performance on the task to create a BPP error score, which was calculated by averaging the percentage of misperceptions and missed images. As we were interested in the neural correlates of misperceptions, we did not use the responses of patients to bistable images in this study.

### Neuroimaging analysis

#### Image acquisition

Imaging was conducted on a 3-T MRI scanner (General Electric). T2*-weighted echo planar functional images were acquired in sequential order with repetition time=3,000 ms, echo time=32 ms, flip angle=90°, 47 axial slices covering the whole brain, field of view=220 mm, and raw voxel size=3.5×3.5×4 mm thick.

### Independent component analysis

After subjecting T2* data to preprocessing (which involved, in order: slice-timing correction; rigid-body realignment using 6 degrees of freedom; strict head-movement repair of scan-to-scan movement of ⩾2 mm using interpolation; normalization to the Echo Planar Image template; and spatial smoothing using an 8-mm Gaussian kernel), images were imported into the GIFT toolbox^3,23^ in SPM8 to perform a group-level spatial independent component analysis (Figure 2). In this study, the group was analyzed as a whole using the InfoMax algorithm to extract 31 maximally independent components, the number of which was estimated from the whole sample using a minimum description length criterion.^3,23^ The components were then spatially sorted at the group level using a set of predefined regions of interest, the co-ordinates of which were taken from previous studies (see Supplementary Table 1 for coordinates).^8–11,24,25^ Based on previous work implicating impaired attentional network communication in visual hallucinations,^1,4,12–15,26^ we chose to extract the default mode network (DMN), the dorsal attention network (DAN), the ventral attention network (VAN), and the visual network (VIS). Spatial maps of each component are presented in Figure 2.

### Network activity

**Figure 1:**
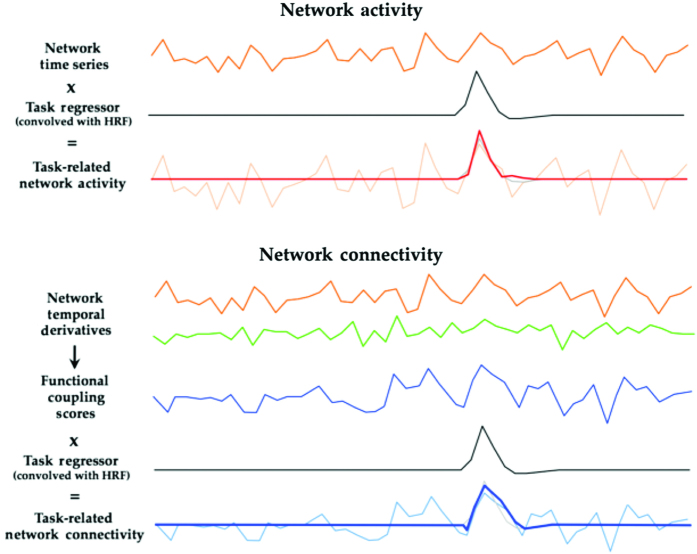
Experimental design. Description of the method using to calculate network activity (top panel) and connectivity (bottom panel). Blood oxygen level-dependent data collected while subjects performed the Bistable Percept Paradigm (BPP) was subjected to independent component analysis, and time series were extracted from each of four networks of interest (shown here in orange). Task regressors modeled on individual subjects’ responses on the task were convolved with the hemodynamic response function and entered into a general linear model with autoregressive modeling, leading to an estimate of network activity for each component. To create an estimate of network connectivity, we first calculated the temporal derivative of each component time course (shown here in orange and green), creating a relative scan-to-scan measure of signal change within each network component. We then multiplied the temporal derivative for each unique pair of measures, leading to a moment-to-moment estimate of functional coupling (shown here in blue). These vectors were then entered into a separate general linear model, allowing an estimate of network connectivity associated with each aspect of the BPP.

Using the Functional Connectivity Toolbox (http://mialab.mrn.org/software), a back-projection method was used to extract the time courses from each component from the ICA analysis, which were then preprocessed further, including de-trending and high-pass filtering (0.009 Hz). The values were then scaled to reflect the percent signal change from the average BOLD intensity within each network component. The task regressors modelling the onset of misperceptions and correct single images were convolved with the canonical hemodynamic response function and then multiplied with the time course of each network component in turn (Figure 1). This resulted in eight unique vectors (four for misperception and four for correct single images) in which positive values represented an increase in activity in a given network associated with a given task condition. When averaged over the course of the experiment for each individual, this allowed for an estimate of the relative amount of network “activity” associated with each outcome from the BPP (Figure 1). These average values were then compared at the group level directly using either independent samples and paired *t*-tests, according to the contrast of interest. Multiple comparisons were controlled using a Bonferroni correction.

### Network connectivity

To create an estimate of network connectivity, we calculated the temporal derivative of each component time course, creating a relative scan-to-scan measure of signal change within each network component (Shine *et al.*, under review). We then multiplied the simple moving average of these temporal derivatives (calculated using a 3-repetition time window in each direction) across the six unique network pairs for each individual, creating a metric that represented the degree of internetwork connectivity in each three-second epoch (Figure 1). Positive scores in this metric imply functional coupling between networks, whereas negative scores imply functional anti-coupling (Shine *et al.*, under review). These time series were then entered into a mixed-effects general linear model (with modeling of autoregressive noise), which allowed for the calculation of a contrast between the parameter estimates for single correct perception and misperceptions. A one-sample *t*-test was then calculated at the group level, with control for multiple comparisons obtained by using a Bonferroni correction.

### Relationship with objective and clinical measures of hallucinations

To determine the presence of a putative relationship between impaired performance on the BPP, the neural correlates of visual misperceptions, and clinical ratings of hallucination severity, we ran two separate statistical analyses in the 33 individuals with at least one misperception on the BPP. First, we ran an ordinary least squares multiple regression analysis investigating the association between the frequency of misperceptions and each of the fMRI outcome measures. *Post hoc* analyses were assessed using Pearson’s product–moment correlations. To determine the individual importance of each network measure, we ran a subsequent analysis in which we first ran a Gram–Schmidt orthogonalization on the network measures before correlating each measure with the severity of misperceptions on the BPP. Finally, we ran separate Spearman’s rank-order correlation analyses to determine whether impairments in network activity and connectivity were associated with worse clinical hallucinations severity. All *α*-values were two-tailed and set to 0.05, and multiple comparisons were controlled for each analysis using a Bonferroni correction.

## Results

Of the 35 individuals in our study, 21 suffered from a high proportion of misperceptions on the BPP (average 42.5±14%; above a previously defined cut score,^1,14^ and were thus defined as “hallucinators” (Table 2). Importantly, each of these individual also displayed hallucinations according to both self-report and objective clinical assessment. In contrast, the remaining 14 subjects displayed low rates of misperceptions (2.8±3%; *t*=8.15, *P*<0.001), similar to those previously reported in age-matched healthy controls (~5%; refs. 1,14), and were thus labeled as “non-hallucinators”. Overall, both groups performed the task effectively, as evidenced by their low rates of “missed” images (Table 2). In addition, there were no significant differences between the two groups on any of the major disease-related variables (*P*>0.100; Table 2), suggesting that the perceptual impairments identified were not driven by other disease-related factors. Consistent with the notion that visual hallucinations exist along a clinical spectrum, we observed strong positive correlations between the rate of misperceptions on the BPP and two independent clinical measures of visual hallucinations (UPDRS Q2: *ρ*=0.733, *P*<0.001; SCOPA–PC_1–4_: *ρ*=0.469, *P*=0.004). In addition, neither the BPP error score nor the frequency of misperceptions correlated significantly with any demographic features of Parkinson’s disease, suggesting that impaired performance on the task was not simply owing to the severity of Parkinson’s disease or dopaminergic medication load.

**Figure 2:**
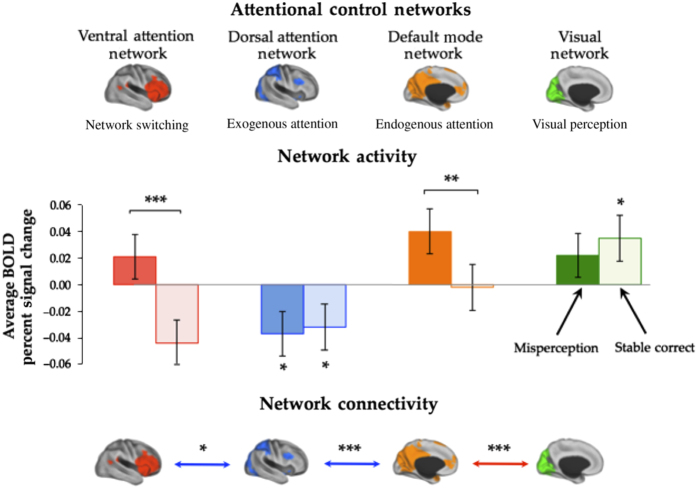
Network activity and connectivity during visual misperceptions in individuals with visual hallucinations. Top panel: Neuroanatomy and putative functions of each of the four networks investigated in this experiment. All results are color coded according to the network of interest: ventral attention network (VAN)—red; dorsal attention network (DAN)—blue; default mode networks (DMNs)—orange; visual networks (VISs)—green. Middle panel: consistent with previous studies,^14,15^ we observed decreased DAN activity during both single correct (*t*=−1.92, *P*=0.034) and misperceived (*t*=−1.86, *P*=0.039) images in individuals with visual hallucinations. We also observed significant increases in the VAN (*t*=2.94, *P*=0.004) and the DMN (*t*=2.22, *P*=0.019) during the comparison of the misperceptions, relative to correct single perception. Lower panel: we observed impaired coupling between the DAN and the VANs (*t*=2.21, *P*=0.034) and DMNs (*t*=3.86, *P*<0.001), along with an increased coupling between the DMNs and VISs (*t*=4.22, *P*<0.001) during misperceptions. Key: dark fill—misperceptions; light fill—stable correct; red arrow—functional coupling; and blue arrow—functional decoupling; *P<0.05, **P<0.01, ***P<0.001.

In the cohort of individuals with Parkinson’s disease, correctly identified “stable” items in the BPP were associated with increased activity within the VIS (*t*=3.20, *P*=0.003), and there were no differences between the two patient groups (*t*=0.84, *P*=0.407). As predicted by our hypothetical framework, we also observed a significant decrease in DAN activity in the group of hallucinators compared with non-hallucinators (*t*=−1.92, *P*=0.034), but no significant differences in the DMNs or VANs (both *P*>0.200), results that are aligned with a previous study.^14^ In the 21 individuals with hallucinations (by definition, the non-hallucinators did not display a high frequency of misperceptions on the BPP), visual misperceptions were associated with significantly increased activity within the VANs (*t*=2.94, *P*=0.004) and DMNs (2.22, *P*=0.019) (Figure 2), a finding aligned with a recent report of abnormal resting state connectivity in individuals with visual hallucinations.^27^ The DAN was also hypoactive during misperceptions (average value: −0.37), but not significantly moreso than during the correct perception of “stable” images (*t*=0.74, *P*=0.469).

We did not observe any significant group-level differences in connectivity during the correct perception of “stable” images. However, when compared with correct “stable” perception in the cohort of 21 hallucinators, misperceptions were associated with multiple abnormal coupling patterns, including an increase in functional coupling between the DMNs and VISs (*t*=4.22, *P*<0.001), along with a decrease in coupling between the DANs and DMNs (*t*=3.86, *P*<0.001), and VANs (*t*=2.21, *P*=0.034; Figure 2). These specific patterns of abnormal connectivity confirm direct predictions of our model,^4,28^ providing evidence to suggest that visual misperceptions in Parkinson’s disease are associated with impaired activity within exogenous attention networks, leading to an over-reliance on endogenous networks in the interpretation of the contents of conscious perception.

To determine whether the group differences reflected the known clinical spectrum of hallucinations in Parkinson’s disease, we performed additional ordinary-least squares multiple regression analyses, in which we related each individuals’ pattern of network activity and connectivity to their individual rate of errors on the BPP. Although the model associated with the frequency of “missed” bistable images on the BPP was not significant (F_10,24_=1.3, *P*=0.272), the frequency of misperceived stable images was strongly significant (*R*=0.77; F_10,24_=3.4, *P*=0.006), suggesting that the significant patterns of impairment were not simply driven by “trait” patterns of network abnormality, but rather were owing to impairments specific to misperception events. *Post hoc* Pearson’s correlations suggested that decreased activity within the DAN (*r*=−0.501, *P*=0.002) impaired connectivity between the DAN and DMNs (*r*=−0.529, *P*=0.001) and the DANs and VANs (*r*=−0.471, *P*=0.004), as well as increased connectivity between the DMNs and VISs (*r*=0.615, *P*<0.001) were the main patterns driving the significant relationship between network abnormalities and visual misperceptions. To delineate the specific contributions of each outcome measure, we performed a Gram–Schmidt orthogonalization of the outcome measures, after which only hypoactivity in the DAN (*r*=−0.494, *P*=0.003) and increased connectivity between the DMNs and VISs (*r*=0.443, *P*=0.007) were significant. All reported results survived strict Bonferroni correction for multiple comparisons.

**Figure 3:**
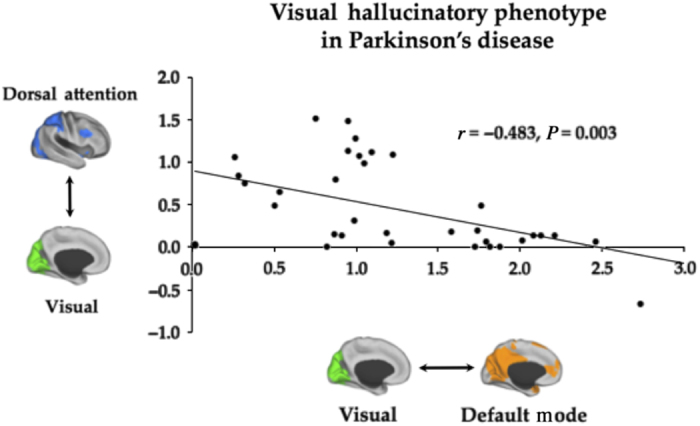
Network connectivity patterns underlying a putative hallucinatory phenotype in Parkinson’s disease. In 35 patients with idiopathic Parkinson’s disease, the extent of impairment in coupling between the dorsal attention network and the visual network (VIS) was strongly predictive of increased coupling between the default mode network and the VIS (*r*=−0.483, *P*=0.003), the latter of which was also strongly correlated with the frequency of visual misperceptions on the Bistable Percept Paradigm (*r*=0.615, *P*<0.001) and the presence of clinical identifiable hallucinations, as measured by question 2 of the Movement Disorders Society-Unified Parkinson’s Disease Rating Scale questionnaire (*r*=0.432, *P*=0.009).

Given the results in the first stage of the experiment, we were interested in interrogating the data for the presence of a potential hallucinatory phenotype. We reasoned that such a relationship would be reflected by patterns of significant connectivity between network activity and connectivity summary statistics across the cohort of 33 subjects (two non-hallucinators with no misperceptions on the BPP were excluded from this analysis). These patterns of “meta-connectivity” showed that the extent of increased connectivity between the DMNs and VISs during misperceptions was significantly correlated with both impairment within the DAN (*r* =−0.528, *P*=0.001), and also with impaired connectivity between the DANs and VISs (*r*=−0.514, *P*=0.001; Figure 3). Therefore, although the DAN was hypoactive during both normal and abnormal perceptions in hallucinators (Figure 2), the extent to which the network was hypoactive was predictive of the strength of connectivity between the DMNs and VISs (Figure 3). Furthermore, we observed a dissociated pattern of connectivity, in which decoupling between the VIS and DAN was correlated with coupling between the VISs and DMNs (Figure 3). Given that each of these outcome measures was strongly correlated with both the frequency of errors on the BPP and independent clinical ratings of hallucination severity, these results provide robust evidence for the hypothesis that attentional network dysfunction is responsible for the pathophysiological mechanism of visual misperceptions in Parkinson’s disease.

To ensure that the patterns of abnormal activity and connectivity associated with misperceptions were indeed related to the clinical presentation of actual visual hallucinations, *post hoc* correlations between significant outcome measures identified from the multiple regression analysis and two independent measures of clinical hallucination severity were conducted: question 2 of the Movement Disorders Society-UPDRS and the SCOPA–PC_1–4_. Each of the measures identified as significant in the multiple regression analysis were significantly correlated with both UPDRS q2 and SCOPA–PC_1–4_ (all *P*<0.01, corrected for multiple comparisons), providing firm evidence that the misperceptions elicited by the BPP are an effective experimental surrogate of visual hallucinations in Parkinson’s disease, and further that the network abnormalities associated by these misperceptions are also strongly related to hallucinations.

## Discussion

Here we provide the first evidence to objectively measure the functional neural correlates of visual misperceptions in patients with Parkinson’s disease and associated clinical visual hallucinations. By comparing misperceptions with normal visual perception, we revealed abnormal patterns of activity and connectivity that were both sensitive and specific to prevalent hallucinations (Figure 2). Specifically, visual misperceptions were associated with the relative inability to recruit exogenous attention systems—namely, the DAN—and a concomitant increase in endogenous systems, comprising the VAN and DMN, the latter of which showed significant functional coupling with the VIS during misperceptions (Figure 2). Importantly, contrary to common models of hallucinations,^5,29^ our data suggest that visual hallucinations are not merely due to aberrant activity within the primary visual system.^4,5,15,29^ Instead, these results directly validate specific predictions from recent models of visual hallucinations in Parkinson’s disease that emphasize the role of attentional network dysfunction^4,15,16^ and provide the first objective estimate of the neuronal architecture responsible for the mechanisms underlying visual misperceptions in Parkinson’s disease.

The misperception events identified during the performance of the BPP were associated with a number of key deficits in neuronal communication. Specifically, visual misperceptions were associated with increased activity within endogenous attention networks (the VANs and DMNs), at the expense of decreased activity within exogenous networks (the DAN; Figure 2). In addition to these patterns of abnormal brain network activity, misperceptions were also associated with impaired connectivity between the exogenous and endogenous networks, however, with a concomitant increase in connectivity between the DMNs and VISs (Figure 2). This result provides evidence to support the notion that activity within the DMN may predispose an individual to hallucinate^14,26^ by allowing the neural regions within the network to pathologically influence ongoing activity within the visual stream,^8,30^ however, only in the context of decreased activity within the DAN.^1,14,19,31–33^ This mechanism could potentially explain the high frequency of pareidolias—the tendency to perceive meaning within ambiguous visual scenes—in individuals with dementia with Lewy bodies, a Parkinsonian syndrome in which individuals suffer from complex visual hallucinations.^27^ Speculatively, unconstrained activity in the DMN during an explicit task may provide an abnormal top-down influence over activity in the temporal lobe subsystem, which would then increase its input to the primary visual system, effectively priming the brain to hallucinate in the absence of appropriate visual input.

In individuals with hallucinations, both veridical and abnormal perceptions were associated with a significant decrease in activity within the DAN (Figure 2), a group of neural regions responsible for a range of exogenous functions, including the refinement of perception of ambiguous stimuli and saccadic eye movements.^14,32^ This result corroborates and extends our previous neuroimaging study,^14^ by showing that, during overt hallucinatory episodes, the decrease in DAN activity is associated with increased activity within endogenous neural networks that are specialized for self-referential thought and introspection, such as the DMNk,^1,4,17,34^ as well as salience monitoring and shifting attention, which are known capacities of the VAN^18,35,36^ (Figure 2). Furthermore, the relative severity of impaired activity within the DAN during misperceptions was also associated with increased connectivity between the DMNs and VISs (Figure 3), further implicating impairments in exogenous attentional mechanisms in the pathophysiology of visual hallucinations.^4,28^ Together, these results suggest that impaired DAN may reflect a predisposing hallucination “trait”, in which transient “state” increases in connectivity between the DMNs and VISs, which would lead to overt hallucinatory episodes.

In a recent study,^37^ we demonstrated key within- and between-network alterations in resting state connectivity that were related to impaired performance on the BPP. Specifically, we observed an increase in connectivity within the VANs and DMNs that scaled with the severity of visual hallucinations, findings that are aligned with the results of our current network activity analysis (i.e., activity at rest). A contrasting pattern of between-network connectivity was observed in our previous resting state study—namely, impaired communication between the VIS with the DANs and VANs—versus those observed during elicited visual misperceptions in the present study –increased connectivity between VISs and DMNs, the latter of which was decoupled from the DANs and VANs. However, there is little consensus in the field regarding the precise role of resting and task-based systems within the human brain. For instance, despite a strong correspondence between the neuronal systems supporting resting state and task-evoked activity in the human brain,^38^ there is emerging evidence that task-related capacities arise owing to targeted patterns of between-network connectivity.^39,40^ Together, this suggests the hypothesis that the network-level abnormalities that predispose an individual to hallucinate (i.e., the “states”) are often not the same systems that are responsible for the actual manifestation of the abnormal behavior (i.e., the “traits”). Future research is required that focuses on these critical issues, both in health and disease.

During the resting state and many cognitive tasks requiring goal-directed behavior, the DMNs and the DANs display an anti-correlated temporal relationship.^20,31^ Although the two networks were not explicitly decoupled during misperceptions elicited by the BPP, patients in this study did show a relative lack of deactivation of the DMN during misperception errors (Figure 2). Indeed, recent research has shown that an inability to effectively quiesce the DMN is associated with poorer performance during exogenous attentional tasks,^1,14,15,31^ and may underlie dysfunction in aging and disease.^31,35,41^ Consistent with our results, these impairments are presumed not to reflect DMN dysfunction *per se*, but rather reduced flexibility in network modulation in the face of changing task demands. It follows that any mechanism that impairs the appropriate “silencing” of the DMN may predispose an individual to hallucinate, perhaps through an increased propensity to display mind-wandering behaviors;^37^ however, hallucinatory symptoms will likely only occur in the context of other pathological processes, such as impaired visual input^42^ or with impairments in exogenous attention.^14^

Although previous studies have attempted to identify the neural correlates of visual hallucinations in Parkinson’s disease, these studies have either attempted to correlate impairments in brain structure^43,44^ or activity with the severity of self-reported hallucinations,^8–11^ or instead drawn inference from impaired performance on tangentially related neuropsychological tasks.^12,13^ One recent study was able to avoid these potential issues and directly explore patterns of hallucinatory behavior by investigating a 66-year-old male with early-stage Parkinson’s disease and a history of persistent, stereotyped hallucinations, while he lay recumbent in an fMRI scanner.^11^ The individual was required to press a button during each hallucinatory episode, effectively alerting the experimenters to moments when he was hallucinating, which they could then compare *post hoc* to the scanned time points without such events. The patient reported 16 such hallucinations during the fMRI scan, and these episodes were associated with widespread increases in activation within the cingulate, insula, frontal lobe, thalamus, and brain stem, with concomitant decreases in activation within occipital, frontomedial, and superior temporal lobes. Despite interesting patterns of overlapping results, there are some important differences between the results of our two studies. However, there are a number of factors that make direct comparison between our experiments potentially problematic. First, case studies in general are notoriously difficult to extrapolate to larger populations, particularly when the individual in question displays an atypical pattern of hallucinations with respect to other individuals with Parkinson’s disease. For instance, the hallucinatory experience of the individual in question was stereotyped, vivid, and scene based, whereas individuals with Parkinson’s disease tend to suffer from relatively minor, object-related misperceptions early in the course of the disease, only losing contact with reality once the disease burden becomes more severe.^4^

Another potential issue with “symptom capture” studies is that the direct comparison of hallucinatory events with time points extracted from an unconstrained portion of the scan necessarily impairs direct interpretability, as one can be less confident of the “baseline” state that events of interest are being compared with. By directly eliciting visual misperceptions in susceptible individuals, the BPP is able to avoid these issues, allowing for the direct assessment of neural activity and connectivity patterns during actual misperception events. Although these episodes differ slightly from the classic definition of hallucinations, which are proposed to occur in the complete absence of sensory input, the presence of strong positive correlations between misperceptions on the BPP and objective clinical measures of hallucinations suggests that the phenomena elicited by the BPP are indeed an effective surrogate for “every day” hallucinations. Regardless of these differences, the extent of the relationship between the frequency of self-reported spontaneous hallucinations and those elicited by experimental means, such as the BPP or the pareidolia test,^27^ is an important question facing the field. Indeed, future studies should be designed not only in an attempt to combine these methods in order to provide a more robust understanding of the pathophysiological mechanism of visual hallucinations in Parkinson’s disease but also in an effort to effectively measure the progression of the symptoms in the clinical setting.

### Conclusion

The results of this study provide the first direct evidence of the abnormalities in neuronal activity and connectivity within the hallucinating brain during elicited visual misperceptions. With evidence from multiple studies converging to support the notion of a common neural mechanism for visual hallucinations irrespective of disease,^4,32^ the path is clear for future studies, which should investigate the precise spatiotemporal mechanisms at the basis of the impairments in attentional flexibility that underlie hallucinations. Although we have shown that Parkinson’s disease can act as an effective neural model for the interrogation of visual hallucinations, it bears mention that there are many other clinical disorders, each with vastly different underlying pathophysiological mechanisms, in which individuals experience hallucinations. Indeed, hallucinatory experiences are actually most commonly reported in the auditory domain, particularly in disorders such as schizophrenia. Interestingly, the results of our analysis are broadly consistent with findings from the schizophrenia literature, in which multiple groups have linked abnormal activity within the DMN to positive symptoms of the condition.^31,41,45,46^ Based on our results, we hypothesize that these alterations in the DMN are likely related to increased connectivity with cortical regions responsible for auditory processing during auditory hallucinations, a proposal consistent with neuroimaging results in schizophrenia.^46^ Regardless, it follows that there is great potential for a trans-diagnostic approach comparing individuals with different disorders that nonetheless share hallucinatory symptoms, which may then lead to the creation of novel therapeutics, with direct clinical benefits across multiple disorders.^47^

## Figures and Tables

**Table 1:** Bistable Percept Paradigm

*Experimental image*	*Image*	*Answer*	*Example response*
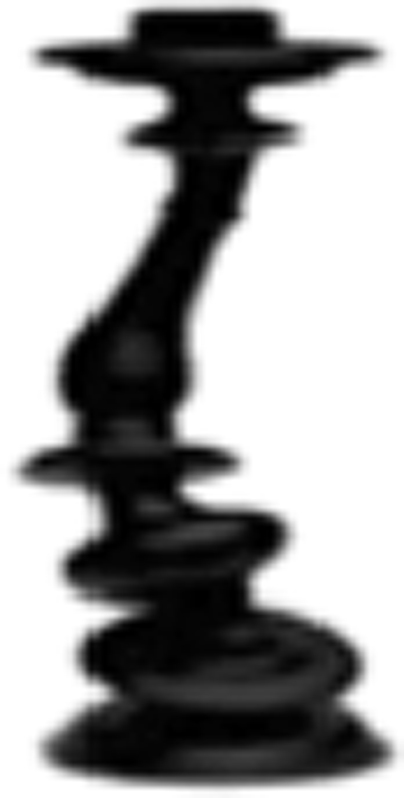	Stable	Single	*“A candlestick”*
		Hidden	*“Faces in the candlestick”*
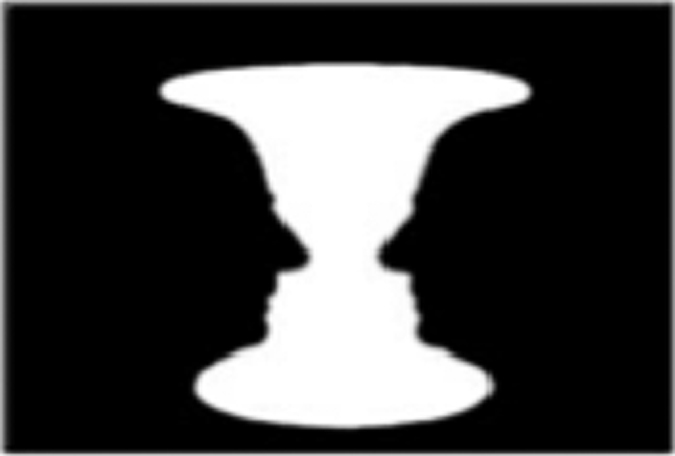	Bistable	Single	*“A candlestick. Nothing else”*
		Hidden	*“Two faces in silhouette and a white candlestick”*
*Example image*	*Subject*	*Answer*	*Subject response*
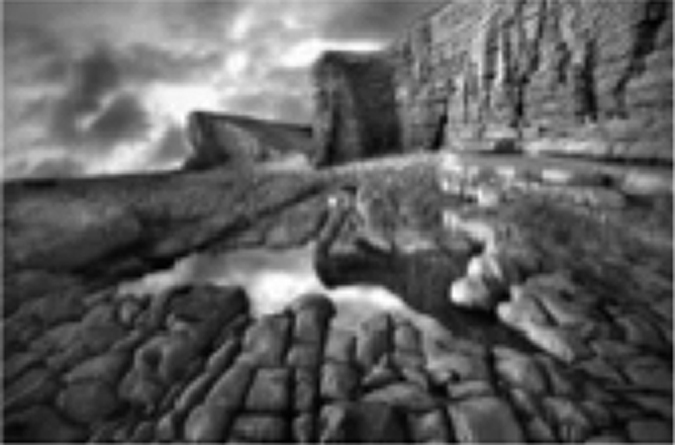	035 (nVH)	Single	*“Rocks and a lake”*
	133 (VH)	Hidden	*“Face on the bottom left hand side of the screen”*
	180 (VH)	Hidden	*“Faces amongst the rocks in the right side of the picture”*
	566 (VH)	Hidden	*“Human figures lying on their backs and faces carved in the stone”*

Abbreviations: nVH, non-hallucinator; VH, hallucinator.

Patients viewed a series of either stable (e.g., black candlestick on white background) or bistable (e.g., white candlestick and black silhouettes of faces) monochromatic images, and were asked to determine whether they perceived a stable (i.e., a single image) or bistable image (i.e., a ‘hidden’ image). Patients responses were coded as either a correct single, a correct hidden, a missed image, or a misperception, with the latter scenario providing an estimation of a visual hallucination. The bottom panel contains a separate example of a stable image (a rocky vista with a lake in the centre), along with four answers that were given by individual subjects in our study).

**Table 2:** Demographics

	*Hallucinators*	*Non-hallucinators*	P *value*
*N*	21	14	
Age	69.3±6	66.3±5	NS.
% Male	67%	71%	NS
Disease duration, years	6.0±3	4.7±4	NS
Hoehn and Yahr, stage	2.2±1	2.1±1	NS
UPDRS, total	40.3±9	34.9±8	NS
DDE, mg/day	960.2±571	1,116.3±609	NS
MoCA	27.2±2	28.6±2	NS
BDI-II	14.1±11	18.9±11	NS
SCOPA–PC_1–4_	2.5±2	0.0±0	<0.001
UPDRS q2	1.6±1	0.0±0	<0.001
BPP error score, %	19.9±7	7.4±3	<0.001
BPP missed images%	11.7±5	10.4±8	NS
BPP misperceptions, %	28.1±9	4.3±5	<0.001
RT—misperceptions, s	6.6±2	6.4±2	NS
RT—single correct, s	6.7±2	6.3±2	NS

Abbreviations: BDI-II, Beck Depression Inventory-II; BPP, Bistable Percept Paradigm; DDE, dopamine dose equivalent; MoCA, Montreal Cognitive Assessment; NS, not significant; RT, reaction time; SCOPA–PC_1–4_, Scales for Outcomes in Parkinson’s Disease–Psychiatric Complications; UPDRS, Unified Parkinson’s Disease Rating Scale Motor sub-score.
