# Supplementary material for: Abnormal connectivity between the default mode and the visual system underlies the manifestation of visual hallucinations in Parkinson’s disease: a task-based fMRI study
Source: NPJ Parkinsons Dis. 2015 Apr 22;1:15003–. doi: 10.1038/npjparkd.2015.3 (PMC5516559; doi:10.1038/npjparkd.2015.3)
Supplement: Supplementary Table 1 [file npjparkd20153-s1.doc]

**Table S1 – ROI Co-ordinates for ICA components**

The most significant coordinates (in MNI space) for each network extracted from the spatial sorting stage following the independent component analysis.

| **Network** | **Hemi** | **Label** | **X** | **Y** | **Z** |
| --- | --- | --- | --- | --- | --- |
|  |  |  |  |  |  |
| *DAN* |  |  |  |  |  |
|  | BL | SPL | +/-30 | -53 | 52 |
|  | BL | FEF | +/-31 | -14 | 54 |
|  | BL | Ling | +/-33 | -85 | -17 |
|  | BL | DLPFC | +/-51 | 14 | 34 |
|  |  |  |  |  |  |
| *DMN_CORE_* |  |  |  |  |  |
|  | Mid | PCu | 0 | -73 | 40 |
|  | BL | Ang | +/-45 | -67 | 40 |
|  | Mid | mPFC | 0 | 53 | -5 |
|  | BL | SFG | +/-42 | 23 | 46 |
|  |  |  |  |  |  |
| *DMN_MTL_* |  |  |  |  |  |
|  | BL | TempP | +/-39 | 11 | -26 |
|  | BL | PHG | +/-21 | -19 | -26 |
|  |  |  |  |  |  |
| *VAN* |  |  |  |  |  |
|  | BL | AI | +/-51 | 14 | -8 |
|  | Mid | dACC | 0 | 11 | 37 |
|  | Mid | PCu | 0 | -55 | 64 |
|  |  |  |  |  |  |
| *VIS* |  |  |  |  |  |
|  | BL | V1 | +/-6 | -94 | 4 |
|  | BL | Calc | +/-21 | -64 | 7 |
|  |  |  |  |  |  |

*Abbreviations:* AI, anterior insula; Calc, calcarine fissure; dACC, dorsal anterior cingulate cortex; DAN, dorsal attention network; DMN, default mode network; FEF, frontal eye fields; Ling, lingual gyrus; mPFC, middle prefrontal cortex; MTL, medial temporal lobe; PCu, precuneus; PHG, parahippocampal gyrus; SPL, superior parietal lobule; TempP, temporal pole; V1, primary visual cortex; VAN, ventral attention network; VIS, visual network.
